# Ambient Air Pollution and Semen Quality in China: A Nationwide Case-Control Study of 27,014 Males with Biomarker-Confirmed Semen Pathology

**DOI:** 10.3390/toxics13040322

**Published:** 2025-04-20

**Authors:** Jianfeng Liu, Zhixiang Fang, Dongyue Chai, Zhipeng Zhu, Qunshan Shen, Xiaojin He

**Affiliations:** 1 State Key Laboratory of Information Engineering in Surveying, Mapping and Remote Sensing, Wuhan University, Wuhan 430070, China; jianfengliu@whu.edu.cn (J.L.);; 2 School of Medicine, Shanghai Jiao Tong University, Shanghai 200080, China; 3 Reproductive Medicine Center, Department of Obstetrics and Gynecology, The First Affiliated Hospital of Anhui Medical University, Hefei 230031, China; 4 NHC Key Laboratory of Study on Abnormal Gametes and Reproductive Tract, Anhui Medical University, Hefei 230032, China; 5 Human Sperm Bank, Department of Obstetrics and Gynecology, The First Affiliated Hospital of Anhui Medical University, Hefei 230022, China

**Keywords:** air pollutants, semen quality, environmental exposure, multicenter study

## Abstract

Amidst China’s rapid industrialization and deteriorating air quality, emerging evidence suggests a parallel decline in male reproductive health. However, large-scale assessments of pollution-semen quality associations remain scarce. This nationwide multicenter study investigated these relationships among 27,014 Chinese men using high-resolution satellite-derived exposure estimates (PM_2.5_, PM_10_, NO_2_, O_3_, CO, and SO_2_) and generalized linear mixed models (GLMM), adjusting for key demographic confounders. A case-control study involving 5256 cases and 21758 controls used the exposure values of air pollutants 90 days prior to sperm collection for epidemiological exposure analysis reactions to obtain the association between sperm quality and air pollution. This study demonstrates significant associations between increased exposure to regional air pollutants and the risk of substandard semen quality in China. Key findings reveal NO_2_’s potential reproductive toxicity, showing a 79.7% increased risk of semen volume abnormalities per 11.34 µg/m^3^ exposure (OR = 1.797, 95% CI: 1.402–2.302). Susceptibility disparities emerged, with 16.4-fold greater PM_2.5_ sensitivity in obese individuals (OR = 1.121 vs. 1.007) and 133% higher PM_10_ risk in urban residents (OR = 1.342 vs. 1.006). Strikingly, SO_2_ exposure at 15% of the WHO 24 h average guideline (6.16 µg/m^3^) was associated with a 3.8% increase in abnormalities, indicating the challenge of the current safety thresholds. These findings highlight the need for policy reforms, including (1) incorporating reproductive health endpoints into air quality standards, (2) implementing antioxidant interventions for high-risk groups, and (3) strengthening traffic emission controls in urban planning. This study underscores the need for comprehensive strategies to mitigate the impact of air pollution on male reproductive health.

## 1. Introduction

Globally, infertility constitutes a major reproductive health burden, affecting an estimated 48 million couples, with prevalence rates ranging from 8% to 12% across different regions [1]. In this context, male factor infertility has emerged as a critical component, accounting for 40–50% of cases through mechanisms predominantly involving compromised semen parameters, including reduced sperm concentration, impaired motility, and abnormal morphology [1,2]. Contemporary population-based studies in developed nations reveal particularly concerning patterns: longitudinal data from European and North American cohorts indicate that 25–30% of young males aged 18–25 years exhibit semen quality metrics below the WHO reference standards, with 15% demonstrating severe oligozoospermia (< 5 million/mL) [2,3]. This secular decline in male reproductive capacity has been systematically documented in multinational registry analyses. Meta-analytic evidence synthesizing 185 studies (1973–2018) demonstrated a 52.4% decrease in global sperm concentration (95% CI: −58.8% to −45.3%), translating to an annual decline rate of 1.06% (*p* < 0.001) [4,5,6]. Regional analyses have revealed accelerated deterioration in Western industrialized regions, where mean sperm counts plummeted from 113.2 million/mL (1973) to 61.3 million/mL (2018), a 54% reduction exceeding global averages [2,5]. Parallel findings from Asian and South American cohorts confirm this transnational pattern, with recent studies in China showing 21% lower sperm concentrations in 2015–2020 than in 2001–2005 (*p* = 0.003) [7,8,9,10]. Epidemiological models estimate that if current trajectories persist, median sperm counts could fall below 40 million/mL by 2050, a threshold associated with exponential increases in time-to-pregnancy and assisted reproduction demand [1].

This biological phenomenon may serve as a critical sentinel indicator of environmental health. Emerging evidence suggests that air pollutants may contribute to adverse reproductive outcomes via potential mechanistic pathways. These pollutants include ozone (O_3_), carbon monoxide (CO), nitrogen dioxide (NO_2_), sulfur dioxide (SO_2_), and particulate matter with diameters less than 2.5 μm and 10 μm (PM_2.5_ and PM_10_). Studies have shown that these pollutants impair sperm DNA integrity. They also reduce sperm concentration, volume, and viability [11,12]. The relationship between air pollutant exposure and diminished sperm quality exhibits considerable regional variations [13,14,15]. While existing evidence confirms the detrimental effects of atmospheric pollutants on sperm parameters within specific urban contexts [16,17,18,19], the comprehensive impact of air pollution on semen quality across China’s male population remains undetermined.

Therefore, it is necessary to conduct a large-scale multicenter study to determine the degree of impact of air pollutants on sperm quality. Current research limitations stem from two key factors: the absence of large-scale, multicenter studies utilizing near-natural population samples regarding male reproductive health and the spatial constraints inherent in conventional air pollution assessment methods. The application of remote sensing technology provides the possibility of accurately obtaining air quality data on a large scale, thereby offering robust data support for monitoring extensive sample populations. The integration of multi-spectral remote sensing platforms has revolutionized large-scale air quality surveillance through the synergistic retrieval of columnar aerosol optical depth, nitrogen dioxide (NO_2_) tropospheric vertical column density, and particulate matter (PM_2.5_) ground-level concentrations at 1 km^2^ spatial resolution [20]. This geospatial monitoring paradigm enables hourly updates of air pollution matrices across continental-scale domains, effectively bridging the critical gap between sparse ground monitoring networks (typically one station per 500–1000 km^2^ in developing regions) and population exposure assessment requirements [21]. Consequently, this technology plays a pivotal role in enhancing our understanding of the impact of air quality on public health and guiding the development of targeted interventions to mitigate the adverse effects of air pollution.

The biological mechanisms through which air pollutants impair semen quality are multifaceted and complex. One of the primary pathways involves the induction of oxidative stress. Airborne pollutants, such as particulate matter (PM), nitrogen dioxide (NO_2_), and ozone (O_3_), generate reactive oxygen species (ROS) within the body. Excessive ROS production can overwhelm the body’s antioxidant defenses, leading to oxidative damage in the sperm cells [5,6,7,8]. This damage manifests in various forms, including mitochondrial dysfunction, lipid peroxidation of cell membranes, and DNA fragmentation in germ cells, all of which can impair spermatogenesis and reduce sperm quality. Additionally, air pollutants can disrupt the hypothalamic-pituitary-gonadal (HPG) axis, which regulates the production and secretion of reproductive hormones. For instance, exposure to pollutants like sulfur dioxide (SO_2_) and carbon monoxide (CO) has been linked to altered levels of testosterone and other hormones critical for sperm production [7,9,10]. Furthermore, environmental stressors induced by air pollution may trigger compensatory physiological responses that adversely affect sperm development [5]. These mechanisms collectively highlight the potential for air pollution to exert both direct and indirect effects on male reproductive health, underscoring the need for further research to elucidate these pathways and their implications for fertility.

Considering the severe decline in semen quality in China [9], this large, multicenter nationwide study was conducted to evaluate the relationship between air pollution and semen quality, involving 27,014 men from 31 provinces. The primary objectives were to quantify the associations between exposure to specific air pollutants (PM_2.5_, PM_10_, NO_2_, O_3_, CO, and SO_2_) and semen quality parameters in a geographically diverse population, identify vulnerable subgroups (e.g., obese individuals and urban residents) with heightened susceptibility to the adverse effects of air pollution, and provide evidence-based recommendations for policy interventions aimed at mitigating the impact of air pollution on male reproductive health. The findings of this extensive research are expected to provide valuable insights into the complex interplay between air pollution and male fertility, guiding future interventions and policies to safeguard public health.

## 2. Materials and Methods

### 2.1. Study Design

We conducted a nationwide multicenter sperm bank-based case-control study to investigate the association between air pollution exposure and spermatogenic dysfunction. The study design comprised 3 key components: (1) rigorous case-control selection and exclusion criteria, (2) air pollution exposure assessment, and (3) analysis of associations while adjusting for potential confounders using generalized linear mixed models (GLMM) to evaluate the effect levels of different pollutants. The cases and controls were selected from the same sampling period across all 10 participating sperm banks.

The following inclusion criteria were applied:

1. Age between 20 and 45 years;
2. No personal or family history of genetic diseases or psychiatric disorders;
3. No infectious diseases (e.g., tuberculosis, various types of hepatitis) or sexually transmitted diseases;
4. No substance abuse, including illicit drugs or chronic alcoholism;
5. No long-term exposure to radiation or toxic/hazardous substances;
6. No systemic or severe organic diseases (e.g., cardiovascular disease or diabetes) present;
7. Abstinence period of 2 to 7 days;
8. No incorrect or missing data;
9. Not Prolonged absence from the residence location for 90 days prior to sperm collection.

From an initial pool of applicants, 27,014 participants were ultimately enrolled in the study, comprising 21,758 individuals with qualified semen quality and 5256 unqualified individuals. Sensitivity analyses targeting specific subpopulations were performed to validate the robustness of our conclusions, confirming the consistency and reliability of our findings across demographic and exposure strata. The study was conducted in accordance with the Declaration of Helsinki and approved by the Ethics Committee of the First Affiliated Hospital of Anhui Medical University (PJ2013-04-12).

### 2.2. Study Population

This case-control study involved 27,014 male donors from 10 sperm banks in China who underwent sperm collection between 1 January 2019 and 31 May 2022.

Participants were recruited from 10 sperm banks located in diverse geographic regions across China, which serve individuals seeking fertility evaluation and those who participate in sperm donation programs. The sperm banks included those in Shanghai, Henan, Guangdong, Shaanxi Sperm Bank, Chongqing, Anhui Sperm Bank, Xinjiang, Hainan, Inner Mongolia, and Jilin. These sperm banks were strategically selected to capture variations in air pollution levels, climate, and socioeconomic factors across different regions of China. At each sperm bank, trained staff administered standardized questionnaires to collect detailed information on the participants’ demographic characteristics, lifestyle factors, and medical history. Semen samples were collected and analyzed on-site using standardized protocols based on the World Health Organization (WHO) guidelines to ensure accuracy and reliability. The use of multiple sperm banks allowed us to recruit a large and diverse sample of participants, enhancing the generalizability of our findings. To ensure a diverse and representative sample, the large sample size (27,014 participants) and geographic diversity of the sperm banks enhanced the external validity of our findings.

Cases were defined as individuals with biomarker-confirmed semen pathology, assessed using the standardized World Health Organization (WHO) criteria. Controls were individuals with normal semen parameters based on standardized assessments.

Recruitment was conducted under the premise of a general semen quality study, which aimed to evaluate factors influencing semen health. This approach ensured that both individuals with and without suspected fertility issues were included in the study. To mitigate potential selection bias, we actively recruited participants from sperm donation programs, who typically have no prior concerns about fertility and represent a population with generally good reproductive health.

After being informed of the content and purpose of this study and signing the consent form, the enrolled participants were interviewed to collect detailed data on their residential address, age, days of abstinence, and date of donation.

Data were collected through face-to-face interviews conducted by trained staff at participating sperm banks. Standardized questionnaires were administered to gather detailed information on demographic characteristics, lifestyle factors, medical history, and other relevant covariates. Semen samples were collected and analyzed on-site using standardized protocols to ensure consistency and accuracy in the assessment of semen quality. This approach allowed for the immediate clarification of any questions and minimized missing or ambiguous responses, ensuring high-quality data collection.

The cases and controls were matched to ensure comparability between groups. Matching was performed based on age (±2 years), geographic region (urban vs. rural), and other demographic factors, including education level (categorized as high school or below, college, or postgraduate), and income level (categorized as low, medium, or high). The geographic region was determined using the participants’ residential addresses, which were linked to satellite-derived air pollution exposure estimates. Additionally, we matched the season of semen sample collection (spring, summer, autumn, or winter) to account for potential seasonal variations in air pollution levels. The matching process was conducted using a propensity score algorithm to minimize confounding factors and ensure balanced distributions of key covariates between cases and controls. This approach enhanced the validity of our findings by reducing the influence of extraneous factors on observed associations. This study followed the Strengthening the Reporting of Observational Studies in Epidemiology (STROBE) reporting guidelines [22].

### 2.3. Semen Analysis

Semen samples were collected by masturbation and deposited in sterile containers in the semen collection room. The samples were then liquefied in a water bath maintained at 37 °C for 60 min. Semen quality parameters, namely semen volume, semen concentration, and progressive motility (PR), were evaluated in accordance with the World Health Organization’s fifth edition of the Laboratory Manual for the Examination and Processing of Human Semen [23]. According to WHO standards, semen is considered qualified if it meets the following criteria: (1) semen volume exceeding 1.5 mL, (2) semen concentration exceeding 15 million per milliliter, and (3) PR exceeding 32%. Based on these criteria, semen quality, semen volume, semen concentration, and PR were dichotomized and labeled as “0” (unqualified) and “1” (qualified) for the subsequent analyses. As illustrated by Equation (1), SC, SV, and PR denote the status of the semen volume, semen concentration, and PR, respectively, for each case. Specifically, SC, SV, and PR are assigned values of “0” for cases with unqualified indicators and “1” for cases with qualified indicators. In addition, X, Y, and t represent the longitude, latitude, and time of each case, respectively.

(1) Cases={C1,C2,…,C27014}={(SQ, SC, SV, PR, X, Y, t)},

### 2.4. Exposure Assessment of Air Pollutants

Given that the spermatogenesis cycle spans 90 days, we utilized the average air pollutant exposure levels from the 90 days preceding sample collection as exposure values [2]. Then, we estimate the exposure to air pollutants(O_3_, CO, NO_2_, SO_2_, PM_2.5_, PM_10_). In the present study, the residential locations of the donors were used as coordinates to evaluate their daily environmental exposure. Their residential locations were geocoded using Baidu Maps to obtain latitude and longitude [24]. As illustrated in Equation (2), the geographical locations X, Y, and time T of each case are associated with 10 environmental exposure values. A sample of data is provided in Table S1.

(2) $$Exposure|(X,Y,t)=\{CO,\;{SO}_{2},\;{NO}_{2},\;O_{3},\;{PM}_{2.5},\;{PM}_{10}\},$$

This study utilized the CHAP (China High-resolution Air Pollutant) dataset for air pollutant exposure assessment from the National Earth System Science Data Center (https://www.geodata.cn, accessed on 19 January 2025). The CHAP dataset integrates multi-source satellite remote sensing, ground observations, atmospheric reanalysis, emission inventories, and model simulations to provide near-surface pollutant concentrations throughout China. PM_2.5_, PM_10_, O_3_, NO_2_, SO_2_, and CO were analyzed using this dataset to support air quality management [25,26,27,28,29]. The CHAP dataset provides spatially continuous air quality estimates across mainland China at a 1 km resolution, integrating multi-source satellite observations (e.g., MODIS and TROPOMI), ground monitoring networks, and atmospheric models. It covers pollutants with daily updates since 2000 and is validated against ground stations (PM_2.5_ R^2^:0.82–0.91; O_3_ RMSE: 8.3–12.7 μg/m^3^). The dataset’s machine learning-based fusion approach enables precise exposure assessment for epidemiological studies, particularly in linking air pollutants to health outcomes like male fertility.

### 2.5. Basic Statistical Analysis

Basic information on donors, including age, abstinence days, type of residence, family income, smoking, BMI, collection month, and sperm pool location,, was collected, and the χ^2^ test was used to screen covariates.

To determine the distribution of semen parameters for the donors, the mean values, standard deviations, minimum values, maximum values, and quartiles of the semen parameters for all semen donors and qualified semen donors were calculated. The Mann−Whitney U test was used to test for significant differences between qualified sperm donors and all other donors [30]. The normal distribution of semen parameters was tested by calculating skewness and kurtosis using the Kolmogorov−Smirnov and Shapiro−Wilk tests [31].

Air pollutant concentrations were compared with the WHO Global Air Quality Guidelines 2021 (AQG2021) [32]. In the AQG2021 standard, all air pollutants are 24 h averages, except for O_3_, which is a single-day maximum eight-hour average value.

### 2.6. Evaluate Effect Levels of Air Pollutants on Semen Quality

To quantify the exposure-response relationships between air pollutant exposure and semen quality, we implemented a generalized linear mixed model (GLMM) with a binomial distribution and logit link function [33]. To avoid collinearity, this study adopted a single-pollutant model and controlled for the levels of other pollutants. This approach accounted for both the fixed effects of air pollutant exposure and the random effects of individual-level covariates. Semen quality parameters (semen quality, volume, concentration, and progressive motility) were dichotomized into binary outcomes (0/1) based on the World Health Organization reference thresholds. Age, abstinence days, type of residence, family income, smoking, BMI, collection month, and sperm pool location were identified as covariates.

The odds ratio (OR) was used to weigh the risk of changes in semen quality parameters due to an IQR increase in air pollutant exposure. The odds ratios (ORs) and their corresponding 95% confidence intervals (CIs) were calculated for the analyses. The OR represents the odds of having semen pathology (cases) compared to normal semen parameters (controls) for each unit increase in air pollutant concentration (e.g., µg/m^3^ for PM_2.5_).

To understand the differential effects of mean exposure versus peak exposure on semen quality, we incorporated the maximum pollution values into our analysis. For each air pollutant (PM_2.5_, PM_10_, NO_2_, O_3_, CO, and SO_2_), we calculated the maximum exposure levels over the study period and compared their associations with semen parameters to those of the mean exposure. This is shown in Equation (3). η is the connection function, and a binary logic function was selected in this study as follows: y represents the classified semen volume, semen concentration, and PR according to the WHO standards of 0 or 1. X is a fixed factor variable, including air pollutants; β is the fixed factor coefficient; Z is the random factor variable, including abstinence days, age of donors, and collection season; u is the random factor coefficient; and ε is the residual term.

(3) η=g(y)=Xβ+Zu+ε,

To assess the robustness of the findings, we conducted sensitivity analyses by stratifying the study population into subgroups based on body mass index (BMI) and residential location. Specifically, we analyzed the exposure-response relationships of air pollutants in individuals with obesity (BMI > 24) and those residing in urban areas. These subgroups were chosen to explore potential effect modification by obesity and urban living, which may influence the relationship between air pollution and semen quality due to differences in metabolic health, lifestyle factors, and exposure levels. Sensitivity analyses were performed using generalized linear mixed models (GLMM), with adjustments for age, education, income, smoking status, and other relevant covariates. Odds ratios (OR) and 95% confidence intervals (CIs) were calculated for each subgroup to evaluate the consistency and strength of the associations.

In addition, we expanded the multi-factor analysis to examine the combined effects of residence (urban vs. rural) and obesity status (obese vs. non-obese) on the association between air pollution and semen quality. Specifically, we stratified the participants into four groups: Urban + Obese, Urban + Non-Obese, Rural + Obese, and Rural + Non-Obese, and analyzed the differential impacts of air pollution within each group.

To explore the localized effects of air pollution on semen quality, we conducted additional analyses stratified by province. We conducted a region-level analysis for six provinces/municipalities (Shanghai, Henan, Guangdong, Shaanxi, Chongqing, and Anhui) with more than 1000 cases. Odds ratios (OR) and 95% confidence intervals (CIs) were compared across provinces to identify regional differences in the association between air pollution and semen quality. This analysis provides insights into how geographic variations in environmental and demographic factors may influence the observed effects.

All statistical analyses were performed using SPSS version 22.0, and statistical significance was considered at a two-sided significance level (*p* < 0.05).

## 3. Results

### 3.1. Basic Information of Donors

**Figure 1:**
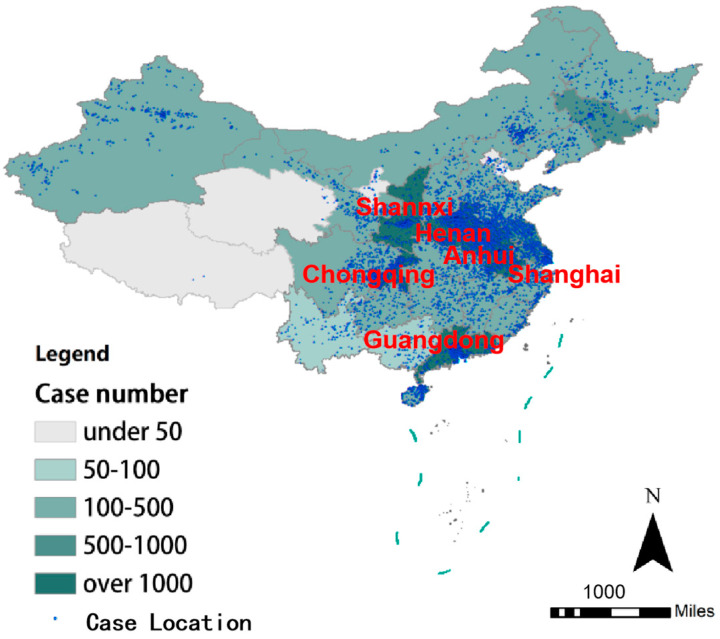
Distribution map of the donor cases.

Samples were gathered from 10 sperm banks across the country, geographically covering all 31 administrative regions of mainland China, as shown in Figure 1. The sample included at least 20 occupations, including students, company employees, and workers, as shown in Table A1. The age range was 20–45 years, with an average age of 27.4 years. Individuals are close to the natural distribution of men of reproductive age, indicating a high level of representation.

Basic information on the 27,014 individuals is displayed in Table 1. Table 1 compares the demographic, behavioral, and geographic characteristics of qualified sperm donors (n = 21,758) and unqualified individuals (n = 5256), with statistical significance assessed using chi-square tests. The key variables include age, abstinence duration, residence type, income, smoking status, BMI, collection month, and sperm bank location. All variables showed highly significant associations (*p* < 0.001) with qualification status, indicating systemic differences between the groups.

The analysis reveals a strong association between age and donor qualification (χ^2^ = 1452.3, *p* < 0.001). Younger men aged 20–24 years are overrepresented in the unqualified group (54.97% vs. 43.70% in qualified), whereas older cohorts (30+ years) show higher qualification rates. For instance, 30–34-year-olds account for 19.28% of qualified donors but only 6.05% of the unqualified individuals. This suggests that age-related biological and behavioral factors influence sperm quality.

Abstinence days are significantly correlated with qualification status (χ^2^ = 230.5, *p* < 0.001). Longer abstinence periods (e.g., 5 days: 37.40% unqualified vs. 30.93% qualified) are linked to higher disqualification rates, potentially reflecting declining sperm motility or DNA fragmentation with prolonged abstinence. Conversely, shorter abstinence (e.g., 7 days: 9.55% unqualified vs. 19.20% qualified) is associated with better outcomes, supporting clinical guidelines for optimal abstinence windows.

Residence type (χ^2^ = 2832.7) and income (χ^2^ = 5669.4) showed stark disparities. Rural residents constituted 68.07% of the unqualified group (vs. 32.56% qualified), likely due to limited healthcare access. Lower-income individuals (≤100,000 CNY) dominate the qualified cohort (73.70% vs. 17.68% unqualified), while “unregistered” income status is associated with disqualification (59.49% unqualified). These patterns underscore the socioeconomic determinants of reproductive health.

Smoking (χ^2^ = 1024.6) and BMI (χ^2^ = 3189.8) are critical predictors. Smokers are disproportionately unqualified (34.42% vs. 19.00% qualified), aligning with evidence of tobacco’s harm to sperm DNA. Similarly, underweight (<18.5 BMI) and overweight (>24 BMI) individuals face elevated disqualification risks (combined 64.33% unqualified vs. 27.70% qualified), emphasizing the role of metabolic health in fertility.

The collection month (χ^2^ = 564.2) and sperm bank location (χ^2^ = 1237.9) highlighted the spatiotemporal influences. Disqualification peaks occur in March (13.51% unqualified) and June–July (~11% unqualified). Geographically, provinces like Jilin (3.79% unqualified vs. 0.91% qualified) and Shaanxi (15.33% unqualified) exhibit elevated risks, suggesting regional health inequities or environmental stressors.

### 3.2. Semen Analysis of Donors

Table 2 compares the semen parameters between the two groups: all participants (n = 27,014) and qualified sperm donors (n = 21,758). Metrics include mean values with standard deviations (SD) and percentile distributions (Min, 25th, 50th, 75th, Max) for semen volume, sperm concentration, and progressive motility (PR). Statistical significance (*p* < 0.01) confirmed marked differences between the groups, reflecting the stringent donor selection criteria.

For all participants, the mean semen volume is 3.55 mL (SD = 1.67), with a median of 3.4 mL and a wide range (0–13.3 mL). The 25th–75th percentile (2.5–4.5 mL) captures typical variation, while the minimum of 0 mL suggests potential measurement errors or pathological cases. Qualified donors exhibit a slightly higher mean volume (3.75 mL, SD = 1.57) and narrower range (1.5–13 mL), with a median of 3.5 mL (25th–75th: 2.5–4.6 mL). This indicates that donor screening favors individuals within or above the normal clinical thresholds.

The average sperm concentration in all participants is 57.01 million/mL (SD = 32.42), with a median of 56 million/mL and extreme outliers (Max = 374 million/mL). In contrast, qualified donors show significantly elevated concentrations (mean = 84.22 million/mL, SD = 20.52; median = 60 million/mL). The donor group’s narrower interquartile range (40–77 million/mL) and higher minimum (15 million/mL vs. 0 in all participants) highlight the exclusion of subfertile individuals during selection.

Progressive motility in all participants averages 49.41% (SD = 14.54), with a median of 50% and severe impairment cases (Min = 0%). Qualified donors demonstrated improved motility (mean = 52.06%, SD = 11.73; median = 53%) and a tighter distribution (25th–75th: 43–61%).

The description of the normality test of the semen parameters is shown in Table 3, where the semen volume and semen concentration showed a left bias, while PR values showed a right bias distribution, which was corrected using the Box-Cox transformation. The absolute value of kurtosis after the Box-Cox transformation was less than 2, and the absolute value of skewness was less than 1, which means that the parameters were generally accepted as a normal distribution.

### 3.3. Exposure of Air Pollution

The exposure of air pollutants to all individuals and qualified sperm donors is presented in Table 4. The table provides statistical summaries of air pollution exposure levels for 27,014 Chinese males, measured across six key pollutants. The metrics include minimum (Min), 25th percentile (25th), median, 75th percentile (75th), maximum (Max), interquartile range (IQR), mean, standard deviation (SD), and the WHO’s 2021 Air Quality Guidelines (AQG2021). These values highlight both nationwide trends and localized extremes in air quality, offering critical insights into the environmental health risks for this population.

For all participants, the median PM_2.5_ exposure (31.91 µg/m^3^) exceeds the WHO AQG2021 guideline (15 µg/m^3^) by more than double. The interquartile range (IQR: 19.76–50.98 µg/m^3^) indicates significant variability, with a maximum exposure of 162.52 µg/m^3^. Among qualified sperm donors, the median exposure (26.80 µg/m^3^) is lower but still exceeds the WHO guideline. The IQR (17.78–42.82 µg/m^3^) and maximum exposure (134.89 µg/m^3^) suggest that even this subgroup faces substantial PM_2.5_ exposure.

The median PM_10_ exposure for all participants (62.07 µg/m^3^) is significantly higher than the WHO guideline (45 µg/m^3^), with a wide IQR (45.82–85.37 µg/m^3^) and a maximum exposure of 572.79 µg/m^3^, indicating severe pollution episodes. For qualified sperm donors, the median exposure (50.90 µg/m^3^) is closer to the WHO guideline but still exceeds it. The IQR (41.24–69.15 µg/m^3^) and maximum exposure (475.42 µg/m^3^) highlight the ongoing exposure risks.

The median SO_2_ exposure for all participants (6.16 µg/m^3^) is well below the WHO guideline (40 µg/m^3^), with a narrow IQR (6.25–8.10 µg/m^3^) and a maximum exposure of 68.70 µg/m^3^. Among qualified sperm donors, the median exposure (5.30 µg/m^3^) is even lower, with a similar IQR (5.00–6.48 µg/m^3^) and maximum exposure (59.08 µg/m^3^). SO_2_ levels appear to be relatively well-controlled in this population.

The median NO_2_ exposure for all participants (26.63 µg/m^3^) exceeds the WHO guideline (25 µg/m^3^), with an IQR (26.16–37.50 µg/m^3^) and a maximum exposure (104.42 µg/m^3^), indicating significant variability. For qualified sperm donors, the median exposure (22.64 µg/m^3^) is slightly below the WHO guideline, with an IQR (21.97–31.50 µg/m^3^) and maximum exposure (89.80 µg/m^3^).

The median O_3_ exposure for all participants (104.14 µg/m^3^) exceeds the WHO guideline (100 µg/m^3^), with a wide IQR (66.48–139.60 µg/m^3^) and maximum exposure (263.42 µg/m^3^). Among qualified sperm donors, the median exposure (89.56 µg/m^3^) is below the WHO guideline, with an IQR (58.50–120.06 µg/m^3^) and maximum exposure (223.91 µg/m^3^).

The median CO exposure for all participants (0.74 mg/m^3^) is well below the WHO guideline (4 mg/m^3^), with a narrow IQR (0.59–0.77 mg/m^3^) and maximum exposure (3.41 mg/m^3^). For qualified sperm donors, the median exposure (0.59 mg/m^3^) is even lower, with an IQR (0.52–0.69 mg/m^3^) and maximum exposure (3.07 mg/m^3^). CO levels appear to be within safe limits.

### 3.4. Effect Levels of Air Pollutants on Semen Quality

To compensate for the problem of the nonlinear distribution of semen parameters, we analyzed the risk of changing natural environmental exposure thresholds for qualified semen indicators using GLMM to further understand the trend and extent of changes in the mean value and peak value of air pollutants affecting semen indicators; the results are shown in Table 5 and Table 6.

Table 5 demonstrates the significant associations between air pollutant exposure and increased risk of semen parameter abnormalities. All measured pollutants (CO, NO_2_, O_3_, PM_10_, PM_2.5_, and SO_2_) showed statistically significant impacts (*p* < 0.01) on at least two indicators. The most prominent effect was observed with NO_2_ on semen volume (OR = 1.797, 95%CI: 1.402–2.302), indicating a 79.7% increased risk of substandard semen volume per 11.34 μg/m^3^ of NO_2_ exposure. Notably, while PM_2.5_ showed significant effects across all parameters, its effect sizes were relatively modest (OR range: 1.002–1.007), suggesting cumulative long-term harm from exposure to fine particulate matter.

Different pollutants exhibited distinct effect patterns. CO emerged as a broad-spectrum but mild risk factor, showing highly significant effects on semen quality (OR = 1.014), volume (OR = 1.006), concentration (OR = 1.008), and PR (OR = 1.012). In contrast, NO_2_ demonstrated exceptional potency for semen volume impairment (OR = 1.797), more than two times stronger than its effects on other parameters, while showing no significant impact on sperm motility rate (PR: OR = 1.168, 95%CI). This specificity suggests that NO_2_ may primarily damage testicular Sertoli cells rather than directly affecting sperm motility machinery.

Semen volume was the most pollution-sensitive parameter, showing significant associations with NO_2_ (OR = 1.797), PM_2.5_ (OR = 1.006), PM_10_ (OR = 1.005), and SO_2_ (OR = 1.029). Conversely, the sperm motility rate (PR) exhibited relative resilience, showing only weak associations with CO (OR = 1.012) and PM_2.5_ (OR = 1.002). Of particular interest is O_3_’s biphasic effect: while significantly impacting semen quality (OR = 1.013) and PR (OR = 1.006), it showed no statistically meaningful effect on sperm concentration (OR = 1.001), possibly due to stage-specific interference with spermatogenic cell cycle regulation.

Table 6 indicates that peak exposure often exerts more pronounced effects, such as the stronger association between peak NO_2_ exposure and semen volume (OR = 2.102, 95%CI: 1.602–2.758) compared to mean exposure (OR = 1.797, 95%CI: 1.402–2.302), and the significantly higher risks of peak PM_2.5_ exposure on semen quality (OR = 1.012, 95%CI: 1.008–1.016) and concentration (OR = 1.010, 95%CI: 1.006–1.014) than that of mean exposure. These findings suggest that short-term high-level pollution exposure may pose more severe risks to semen quality, highlighting the importance of considering both peak and average exposure levels in future studies to provide a more comprehensive assessment of environmental health risks.

As shown in Table A2, a region-level analysis of the effects of air pollution on semen parameters in Shanghai, Henan, Guangdong, Shaanxi, Chongqing, and Anhui was conducted. The results revealed significant regional variations, with NO_2_ and PM_2.5_ dominating in urbanized areas like Shanghai and Chongqing, while SO_2_ and PM_10_ were more influential in industrial regions such as Henan and Shaanxi. For instance, in Shanghai, peak NO_2_ exposure had the strongest impact on semen volume (OR = 2.305, 95%CI: 1.802–2.948), while in Henan, SO_2_ showed a significant effect on semen concentration (OR = 1.031, 95%CI: 1.018–1.044) Coastal regions like Guangdong showed weaker NO_2_ effects due to better pollutant dispersion, whereas inland regions like Shaanxi exhibited stronger impacts from PM10 and SO_2_, with PM10 significantly affecting semen volume (OR = 1.007, 95%CI: 1.004–1.010).

Table 7 and Table 8 show the associations between exposure to air pollution and semen parameters in the obese population and urban population. The sensitivity analysis results confirmed the impact of air pollution on sperm quality in two specific populations, and the model data showed that obese and urban populations were more sensitive to the risk of poor semen quality caused by exposure to air pollution.

As shown in Table 7, air pollutants demonstrated a markedly stronger association with obese individuals than with the general population (Table 7). PM_2.5_’s association with semen quality surged from OR = 1.007 to 1.121 (*p* < 0.001), translating to a 12.1% increased risk per IQR exposure to pollutants. NO_2_ maintained its dominance in impairing semen volume (OR = 1.870 vs. 1.797), likely exacerbated by adipose tissue-derived pro-inflammatory cytokines (e.g., IL-6), which enhance pollutant toxicity. Strikingly, SO_2_’s association with semen quality intensified by 191% (OR = 1.107 vs. 1.038), suggesting that obesity may impair hepatic detoxification pathways for sulfur compounds.

CO exhibited a paradoxical association: while significantly increasing the risk of PR (OR = 1.054, CI = 1.029–1.079), it showed no meaningful association with semen volume (OR = 1.048, CI = 0.995–1.101). This dichotomy may stem from altered hemoglobin-CO binding kinetics in obesity, where elevated blood volume dilutes carboxyhemoglobin but prolongs its half-life in tissues.

As shown in Table 8, urban residents faced compounded hazards, with PM_10_’s OR for semen quality increasing to 1.342 (*** *p* < 0.001) versus 1.006 in the general population. Ozone (O_3_) demonstrated unprecedented potency (OR = 1.274 for semen quality), likely due to photochemical interactions with vehicular NOx emissions, forming secondary organic aerosols. The NO_2_-PM_2.5_ synergy was particularly alarming; their combined OR product (1.969 × 1.369 = 2.695) far exceeded the individual association, indicating multiplicative damage to spermatogenesis.

Sperm motility (PR) emerged as the most vulnerable parameter in urban settings, with PM_10_ OR = 1.313 (CI = 1.141–1.486) versus 1.005 in Table 5. This aligns with urban-specific exposure to transition metals (e.g., lead and cadmium adsorbed on PM), which disrupts mitochondrial function in the sperm tails. Notably, CO’s effect on PR surged to OR = 1.246 (urban) from 1.012 (general population), implicating chronic exposure to traffic-related ultrafine particles.

As shown in Table A3, we conducted a detailed multi-factor analysis examining the combined associations of residence (urban vs. rural) and obesity status on the associations between air pollution and semen parameters. The analysis revealed significant regional and individual variation. In urban + obese populations, air pollutants like NO_2_ and PM_2.5_ had the strongest negative effects on semen quality and volume, with NO_2_ exposure showing an OR of 1.969 (95%CI: 1.522–2.416) for semen quality and PM2.5 exposure significantly impacting sperm motility rate (OR = 1.288, 95%CI: 1.123–1.454). Similarly, rural + obese populations exhibited amplified effects of PM_10_ and SO_2_, particularly on semen concentration (OR = 1.040, 95%CI: 1.010–1.070 for PM_10_) and volume (OR = 1.050, 95%CI: 1.010–1.090 for PM_10_). In contrast, urban + non-obese and rural + non-obese groups showed greater resilience, with weaker associations between air pollution and semen parameters. For example, in rural non-obese individuals, PM_10_ exposure had a minimal effect on semen volume (OR = 1.020, 95%CI: 0.990–1.050), while CO exposure showed a modest impact on semen quality (OR = 1.050, 95%CI: 1.010–1.090).

These findings highlight the synergistic association between urban residence and obesity, which significantly amplifies the effect of air pollution on semen quality. For instance, in urban obese populations, O_3_ exposure had a strong effect on semen quality (OR = 1.274, 95%CI: 1.234–1.315), while in rural obese populations, SO_2_ exposure significantly impacted semen concentration (OR = 1.060, 95%CI: 1.030–1.090). Conversely, protective factors, such as non-obesity and rural residence, appear to mitigate these risks, likely due to lower systemic inflammation and healthier lifestyles. For example, rural non-obese individuals showed minimal effects of PM_2.5_ on sperm motility rate (OR = 1.030, 95%CI: 1.010–1.050), while urban obese individuals faced much higher risks (OR = 1.288, 95%CI: 1.123–1.454 for PM_2.5_). These interactions underscore the importance of considering both environmental and individual factors when assessing the impact of air pollution on reproductive health.

## 4. Discussion

### 4.1. Strengths and Innovations of the Study

In our study, based on a combination of air pollution and geographic data from a multicenter, region-wide male sperm bank in China, we employed a GLMM to determine the level of influence of air pollution on sperm quality. Compared with previous studies, the current investigation is a national-level, large-scale, and multicenter study covering a diverse range of independent variables and geographic regions. In addition, the study population is representative of a broad range of ages and occupations, which more accurately reflects the natural population of Chinese men of childbearing age. A unique advantage of our study is that we have a wide range of independent variable exposure concentrations, allowing the examination of exposure-response relationships ranging from relatively low doses to toxicologically significant doses. Furthermore, the wide range of remote sensing data and raster products facilitates a more precise estimation of individual environmental exposures, which helps minimize the incorrect assessment of environmental exposures.

### 4.2. Impact of Air Pollution on Semen Quality

Our findings suggest that concurrent exposure to ambient air pollutants during sperm development has a significant effect on semen quality, particularly with respect to semen volume, concentration, and PR. Oxidative stress is a potential biological mechanism that may underlie these associations. Environmental pollutants, such as particulate matter, are known to induce ROS production [34,35]. The resultant redox imbalance can impair spermatogenesis through multiple pathways, including mitochondrial dysfunction in developing sperm, lipid peroxidation of cell membranes, and DNA fragmentation in germ cells [36,37,38]. These findings illustrate the potential for improving semen quality through dual approaches: reducing adverse environmental exposure and implementing antioxidant interventions targeting oxidative stress pathways, particularly during vulnerable periods of spermatogenic development.

Air pollutants may activate the hypothalamic-pituitary-adrenal and hypothalamic-pituitary-gonadal axes [39,40], potentially disrupting the production and secretion of normal male reproductive hormones, which could contribute to a decrease in sperm quality. This study provides robust evidence of significant associations between increased exposure to regional air pollutants and the risk of substandard semen quality in China. Notably, our study demonstrated a significant correlation between NO_2_ levels and semen quality. A previous study found that NO_2_ was associated with reduced normal sperm morphology [41], which is consistent with the findings of this study. This consistency across studies suggests that NO_2_ may be a critical air pollutant contributing to adverse male reproductive health outcomes, even in men without prior fertility issues. Previous studies have shown that exposure to SO_2_ is associated with decreased total motility and PR [42,43], which is consistent with the findings of the present study. Additionally, it is worth noting that the SO_2_ exposure in the present study was overwhelmingly below the recommended value of the WHO Global Air Quality Guidelines (24-h AQG level: 40 μg/m^3^), which suggests that even SO_2_ exposure at either low or high concentrations can cause reproductive toxicity to sperm, which warrants attention in subsequent studies. O_3_ is another air pollutant of noteworthy concern, especially given the discernible improvement of other air pollutants in China, whereas the O_3_ concentration shows a clear upward trend [44]. Exposure to elevated O_3_ levels is closely associated with a heightened risk of oxidative stress, leading to reduced sperm quality. Several studies have shown that O_3_ exposure leads to a decrease in semen concentration and sperm viability, which is consistent with the findings of the present study. In addition, several studies have reported that PM negatively affects semen quality. For example, PM exposure has been associated with reduced sperm concentration and progressive motility and may also reduce total motility [45,46], corroborating the findings of the present study. There is evidence that exposure to PM_10_ and PM_2.5_ affects endocrine and serum hormone levels, thereby negatively affecting male sperm quality.

### 4.3. Public Health Implications and Policy Recommendations

These results have important implications for public health. Firstly, our research highlights that air pollution is associated with adverse effects on semen quality, a crucial determinant of male fertility, warranting further investigation and public health attention. Our study provides evidence that exposure to O_3_, CO, NO_2_, SO_2_, PM_10_, and PM_2.5_ is associated with decreased semen quality, underscoring the importance of controlling air pollutant concentrations in countries with severe air pollution. Secondly, this study provides researchers with information to better understand the possible mechanisms by which natural environmental exposure leads to semen volume, semen concentration, and PR, thus providing data support for the study of the impact mechanism of oxidative stress response on sperm quality in the future.

### 4.4. Limitations

This study has several limitations that should be considered when interpreting the results. The recruitment of participants from sperm banks may have introduced selection bias, as individuals who visit sperm banks may differ from the general population in terms of fertility concerns, health behaviors, or socioeconomic status. To mitigate this, we included participants from both fertility evaluation and sperm donation programs, ensuring a more diverse sample. However, we acknowledge that the sample may still over-represent individuals with greater concerns regarding their reproductive health. Moreover, the voluntary nature of participation may have led to participation bias, as individuals more motivated to contribute to research or those with specific health concerns may have been more likely to enroll. We performed sensitivity analyses by stratifying participants based on their reasons for visiting the sperm bank (e.g., fertility evaluation vs. sperm donation) and found consistent results across subgroups; however, we recognize that these strategies may not fully eliminate the potential for bias. While we adjusted for key demographic and lifestyle confounders in our statistical models, there may still be residual confounding from unmeasured factors, such as dietary habits or genetic predispositions, that could influence the observed associations. Despite these limitations, our study provides valuable insights into the association between air pollution and semen quality, supported by a large, geographically diverse sample.

As a cross-sectional study, our findings are limited in their ability to establish causal relationships between exposure to air pollution and semen quality. While we observed significant associations, unmeasured confounders (e.g., dietary habits and genetic predispositions) or reverse causality (e.g., individuals with poor semen quality may be more susceptible to environmental exposures) could influence the results.

While our study provides robust evidence of the association between air pollution exposure and semen quality, the absence of experimental data on pollutant dosage in biological fluids (e.g., blood or seminal fluid) limits our ability to establish direct causal mechanisms.

### 4.5. Future Work

Future research in this field should aim to address the limitations of the current study and deepen our understanding of the complex relationship between environmental exposure and semen quality. To refine exposure assessment, future studies should integrate personal monitoring devices, collect detailed data on indoor environmental conditions, and account for the influence of occupational exposure and lifestyle factors. Given the substantial amount of time individuals spend indoors, a specific focus on indoor air quality and its impact on semen quality is warranted.

Experimental validation of the biological mechanisms underlying these associations is, therefore, essential. This could include in vitro and in vivo studies to investigate the roles of oxidative stress, mitochondrial dysfunction, lipid peroxidation, and DNA fragmentation in spermatogenic impairment. Additionally, longitudinal studies are needed to assess the long-term effects of environmental exposure on semen quality and reproductive outcomes, providing insights into the temporal relationships and potential causal pathways.

Policy and intervention studies are also critical for evaluating the effectiveness of measures aimed at reducing air pollution and improving environmental quality on reproductive health. Developing and testing interventions targeting oxidative stress pathways could help determine their efficacy in enhancing semen quality and improving reproductive outcomes.

Furthermore, future studies should aim to include broader and more diverse populations, incorporate biomonitoring of pollutants in biological samples, and address potential biases to further elucidate the pathways underlying these associations. By addressing these gaps, future research can provide a more comprehensive understanding of the impact of environmental exposure on male reproductive health and inform effective public health strategies.

## 5. Conclusions

In this study, we aimed to investigate the association between exposure to regional air pollutants and semen quality in a large, geographically diverse population in China. Our findings demonstrate significant associations between increased exposure to air pollutants (e.g., PM_2.5_, NO_2_, and SO_2_) and adverse effects on semen quality parameters, including semen volume, concentration, and motility. Notably, NO_2_ exhibited the strongest reproductive toxicity, with a 79.7% increased risk of semen volume abnormalities per 11.34 µg/m^3^ of exposure. We also identified vulnerable subgroups, such as obese individuals and urban residents, who exhibited heightened susceptibility to the adverse effects of air pollution exposure. These findings underscore the importance of incorporating reproductive health endpoints into air quality standards and highlight the need for targeted interventions to mitigate the impact of air pollution on male reproductive health. Specifically, our results suggest that reducing adverse environmental exposure and implementing antioxidant interventions targeting oxidative stress pathways could improve semen quality, particularly during vulnerable windows of spermatogenic development.

Future research should focus on longitudinal studies to better understand the temporal relationships and potential causal mechanisms underlying these associations. Additionally, incorporating biomonitoring of pollutants in biological samples and exploring the role of indoor air quality may provide further insights into the pathways linking environmental exposure to reproductive health outcomes. By addressing these research objectives, our study contributes to the growing body of evidence on the impact of air pollution on male reproductive health and provides a foundation for future public health interventions and policy reforms.

## Figures and Tables

**Table 1:** Characteristics of the study participants.

Characteristic	Total (n = 27,014)		Qualified (n = 21,758)		Unqualified (n = 5256)	
Age (year) (χ^2^ = 1452.3, *p* < 0.001 **)						
20–24	12398	45.89%	9509	43.70%	2889	54.97%
25–29	7658	28.35%	6072	27.91%	1586	30.18%
30–34	4513	16.71%	4195	19.28%	318	6.05%
35–39	1958	7.25%	1593	7.32%	365	6.94%
40–45	487	1.80%	389	1.79%	98	1.86%
Abstinence days (χ^2^ = 230.5, *p* < 0.001 **)						
2	407	1.51%	319	1.47%	88	1.67%
3	4918	18.21%	3899	17.92%	1019	19.39%
4	6612	24.48%	5263	24.19%	1349	25.67%
5	8696	32.19%	6730	30.93%	1966	37.40%
6	1701	6.30%	1369	6.29%	332	6.32%
7	4680	17.32%	4178	19.20%	502	9.55%
Type of residence (χ^2^ = 2832.7, *p* < 0.001 **)						
Rural	10662	39.47%	7084	32.56%	3578	68.07%
Urban	16352	60.53%	14673	67.44%	1679	31.94%
Family income (χ^2^ = 5669.4, *p* < 0.001 **)						
≤100,000 CNY	16964	62.80%	16035	73.70%	929	17.68%
>100,000 CNY	2636	9.76%	1436	6.60%	1200	22.83%
Unregistered	7414	27.45%	4287	19.70%	3127	59.49%
Smoking (χ^2^ = 1024.6, *p* < 0.001 **)						
No	11076	41.00%	9312	42.80%	1764	33.56%
Yes	5943	22.00%	4134	19.00%	1809	34.42%
Unregistered	9995	37.00%	8312	38.20%	1683	32.02%
BMI (χ^2^ = 3189.8, *p* < 0.001**)						
18.5–23.9	17607	65.18%	15731	72.30%	1876	35.69%
<18.5	3583	13.26%	2480	11.40%	1103	20.99%
>24	5824	21.56%	3546	16.30%	2278	43.34%
Collection month (χ^2^ = 564.2, *p* < 0.001 **)						
January	1562	5.78%	1235	5.68%	327	6.22%
February	1105	4.09%	844	3.88%	261	4.97%
March	3194	11.82%	2484	11.42%	710	13.51%
April	3278	12.13%	2674	12.29%	604	11.49%
May	2514	9.31%	1998	9.18%	516	9.82%
June	2644	9.79%	2057	9.45%	587	11.17%
July	2766	10.24%	2223	10.22%	543	10.33%
August	1872	6.93%	1523	7.00%	349	6.64%
September	1990	7.37%	1605	7.38%	385	7.32%
October	2099	7.77%	1641	7.54%	458	8.71%
November	2034	7.53%	1604	7.37%	430	8.18%
December	1956	7.24%	1870	8.59%	86	1.64%
Sperm pool location (χ^2^ = 1237.9, *p* < 0.001 **)						
Shanghai	7126	26.38%	5957	27.38%	1169	22.24%
Henan	6237	23.09%	5083	23.36%	1154	21.96%
Guangdong	3373	12.49%	2641	12.14%	732	13.93%
Shaanxi	3197	11.83%	2391	10.99%	806	15.33%
Chongqing	2999	11.10%	2429	11.16%	570	10.84%
Anhui	1813	6.71%	1537	7.06%	276	5.25%
Xinjiang	758	2.81%	648	2.98%	110	2.09%
Hainan	636	2.35%	494	2.27%	142	2.70%
Inner Mongolia	479	1.77%	381	1.75%	98	1.86%
Jilin	396	1.47%	197	0.91%	199	3.79%

** Statistical analysis was performed using the chi-square test(*p* < 0.01). The percentage symbol (%) was added to all the proportion values.

**Table 2:** Distribution of semen parameters among participants.

Semen Parameter	Mean (SD)	Percentile				
		Min	25th	50th	75th	Max
All participants (n = 27,014)						
Semen volume (ml)	3.55 (1.67)	0	2.5	3.4	4.5	13.3
Sperm concentration (10^6^/mL)	57.01 (32.42)	0	34	56	74	374
PR * (%)	49.41 (14.54)	0	40	50	60	90
Qualified sperm donors (n = 21,758)						
Semen volume (mL) ^a^	3.75 (1.57)	1.5	2.5	3.5	4.6	13
Semen concentration (10^6^/mL) ^a^	84.22 (20.52)	15	40	60	77	374
PR(%)	52.06 (11.73)	32	43	53	61	90

* PR: Progressive motility. ^a^ indicates significant differences between qualified sperm donors and all participants after the Mann-Whitney U test (*p* < 0.01).

**Table 3:** Description of the normality test for semen parameters.

Semen Parameter	Skewness	Kurtosis	Kolmogorov–Smirnov Test		Shapiro–Wilk Test	
			D Value	*p*	W Value	*p*
Parameters before Box-Cox transformation						
Semen volume	0.873	1.757	0.088	0.000 **	0.961	0.000 **
Semen concentration	0.87	1.869	0.066	0.000 **	0.959	0.000 **
PR	−0.767	0.477	0.127	0.000 **	0.955	0.000 **
Parameters after Box-Cox transformation						
Semen volume	0.011	0.268	0.055	0.000 **	0.996	0.000 **
Semen concentration	−0.041	0.166	0.064	0.000 **	0.992	0.000 **
PR	−0.239	−0.437	0.128	0.000 **	0.982	0.000 **

PR: progressive motility. ** *p* < 0.01

**Table 4:** Distribution characteristics of average air pollutant exposure during 0–90 lag days in all participants and qualified sperm donors.

Air Pollutants	Min	25th	Median	75th	Max	IQR	Mean	SD	AQG2021 ^a^
All participants (n = 27,014)									
PM_2.5_ (μg/m^3^)	4.99	19.76	31.91	50.98	162.52	31.23	38.82	19.88	15
PM_10_ (μg/m^3^)	13.4	45.82	62.07	85.37	572.79	39.55	55.19	30.65	45
SO_2_ (μg/m^3^)	1.73	6.25	6.16	8.1	68.7	1.86	6.38	2.6	40
NO_2_ (μg/m^3^)	4.75	26.16	26.63	37.5	104.42	11.34	34.42	10.99	25
O_3_ (μg/m^3^)	21.69	66.48	104.14	139.6	263.42	73.11	109.49	32.57	100 ^b^
CO (mg/m^3^)	0.11	0.59	0.74	0.77	3.41	0.18	0.65	0.18	4
Qualified sperm donors (n = 21,758)									
PM_2.5_ (μg/m^3^)	4.19	17.78	26.80	42.82	134.89	25.04	32.22	16.30	15
PM_10_ (μg/m^3^)	12.06	41.24	50.90	69.15	475.42	27.91	47.46	25.75	45
SO_2_ (μg/m^3^)	1.51	5.00	5.30	6.48	59.08	1.48	5.42	2.24	40
NO_2_ (μg/m^3^)	4.23	21.97	22.64	31.50	89.80	9.53	30.98	9.67	25
O_3_ (μg/m^3^)	18.44	58.50	89.56	120.06	223.91	61.55	97.45	27.68	100 ^b^
CO (mg/m^3^)	0.09	0.52	0.59	0.69	3.07	0.17	0.58	0.15	4

^a^ AQG2021:WHO Global Air Quality Guidelines 2021. ^b^ In the AQG2021 standard, all air pollutants are 24 h averages, except for O_3_, which is a single-day maximum eight-hour average.

**Table 5:** Associations between exposure to mean values of air pollutants and semen parameters in all participants.

Air Pollutants	Semen Quality	Semen Volume	Semen Concentration	PR
CO	1.014 ***	1.006 ***	1.008 ***	1.012 ***
	(1.012,1.016)	(1.005,1.008)	(1.007,1.009)	(1.011,1.014)
NO_2_	1.797 ***	1.242 **	1.311 **	1.168
	(1.402,2.302)	(1.058,1.458)	(1.042,1.651)	(0.945,1.444)
O_3_	1.013 ***	1.001	1.007 ***	1.006 ***
	(1.008,1.017)	(0.997,1.004)	(1.004,1.011)	(1.003,1.009)
PM_10_	1.006 ***	1.005 ***	1.004 ***	1.005 ***
	(1.005,1.007)	(1.005,1.006)	(1.003,1.006)	(1.004,1.006)
PM_2.5_	1.007 ***	1.006 ***	1.007 ***	1.002 **
	(1.005,1.009)	(1.005,1.008)	(1.005,1.010)	(1.000,1.004)
SO_2_	1.038 ***	1.029 ***	1.012	1.024 ***
	(1.023,1.053)	(1.020,1.039)	(0.999,1.026)	(1.012,1.037)

The values in the table represent OR values, with values greater than 1 indicating an increase in unqualified risk with IQR mean value of air pollutants increase and values less than 1 indicating a decrease in unqualified risk. PR: progressive motility; ** *p* < 0.01 *** *p* < 0.001.

**Table 6:** Associations between exposure to peak value air pollution and semen parameters in all participants.

Air Pollutants	Semen Quality	Semen Volume	Semen Concentration	PR
CO	1.018 ***	1.008 ***	1.010 ***	1.014 ***
	(1.014,1.022)	(1.006,1.010)	(1.008,1.012)	(1.011,1.017)
NO_2_	2.102 ***	1.242 **	1.311 **	1.168
	(1.602,2.758)	(1.058,1.458)	(1.042,1.651)	(0.945,1.444)
O_3_	1.018 ***	1.001	1.007 ***	1.006 ***
	(1.010,1.026)	(0.997,1.004)	(1.004,1.011)	(1.003,1.009)
PM_10_	1.008 ***	1.006 ***	1.005 ***	1.006 ***
	(1.006,1.010)	(1.005,1.008)	(1.003,1.007)	(1.004,1.008)
PM_2.5_	1.012 ***	1.008 ***	1.010 ***	1.003 **
	(1.008,1.016)	(1.006,1.010)	(1.006,1.014)	(1.000,1.006)
SO_2_	1.042 ***	1.031 ***	1.014	1.026 ***
	(1.028,1.056)	(1.022,1.041)	(0.999,1.028)	(1.014,1.039)

The values in the table represent OR values, with values greater than 1 indicating an increase in unqualified risk with an increase in the IQR of the peak value of air pollutants and values less than 1 indicating a decrease in unqualified risk. PR: progressive motility. ** *p* < 0.01 *** *p* < 0.001.

**Table 7:** Associations between exposure to air pollutants and semen parameters in the obese population.

Air Pollutants	Semen Quality	Semen Volume	Semen Concentration	PR
CO	1.143 ***	1.048	1.025 ***	1.054 ***
	(1.080,1.206)	(0.995,1.101)	(1.006,1.044)	(1.029,1.079)
NO_2_	1.870 ***	1.361 ***	1.301	1.405 ***
	(1.428,2.312)	(1.132,1.591)	(0.964,1.638)	(1.090,1.721)
O_3_	1.076 ***	1.050 ***	1.070 ***	1.037 ***
	(1.049,1.102)	(1.012,1.087)	(1.059,1.080)	(1.033,1.042)
PM_10_	1.079 ***	1.075 ***	1.062 ***	1.075 ***
	(1.039,1.118)	(1.002,1.147)	(1.021,1.104)	(1.069,1.082)
PM_2.5_	1.121 ***	1.107 ***	1.122 ***	1.077 ***
	(1.085,1.156)	(1.054,1.160)	(1.051,1.193)	(1.071,1.084)
SO_2_	1.107 ***	1.091 ***	1.086 ***	1.046 ***
	(1.009,1.206)	(1.051,1.132)	(1.071,1.101)	(1.020,1.071)

The values in the table represent OR values, with values greater than 1 indicating an increase in unqualified risk with IQR air pollutant increases and values less than 1 indicating a decrease in unqualified risk. PR: progressive motility. *** *p* < 0.001

**Table 8:** Associations between exposure to air pollutants and semen parameters in the urban population.

Air Pollutants	Semen Quality	Semen Volume	Semen Concentration	PR
CO	1.256 ***	1.223 ***	1.212 ***	1.246 ***
	(1.083,1.428)	(1.135,1.311)	(1.153,1.270)	(1.121,1.266)
NO_2_	1.969 ***	1.617 ***	1.601 ***	1.418 ***
	(1.522,2.416)	(1.295,1.941)	(1.252,1.947)	(1.031,1.806)
O_3_	1.274 ***	1.141 ***	1.235 ***	1.272 ***
	(1.234,1.315)	(1.102,1.181)	(1.153,1.318)	(1.244,1.301)
PM_10_	1.342 ***	1.238 ***	1.307 ***	1.313 ***
	(1.271,1.414)	(1.088,1.387)	(1.158,1.456)	(1.141,1.486)
PM_2.5_	1.369 ***	1.304 ***	1.247 ***	1.288 ***
	(1.209,1.528)	(1.216,1.393)	(1.181,1.313)	(1.123,1.454)
SO_2_	1.337 ***	1.270 ***	1.162 ***	1.296 ***
	(1.266,1.409)	(1.202,1.338)	(1.158,1.166)	(1.050,1.543)

The values in the table represent OR values, with values greater than 1 indicating an increase in unqualified risk with IQR air pollutant increases, and values less than 1 indicating a decrease in unqualified risk. PR: progressive motility. *** *p* < 0.001.

## Data Availability

The data presented in this study are available upon request from the corresponding author for ethical reasons.

## Supplementary Materials

The following supporting information can be downloaded at: https://www.mdpi.com/article/10.3390/toxics13040322/s1, Table S1: Raw Data Sample.

## Abbreviations

WHO	World Health Organization
BMI	Body Mass Index
PM_2.5_	Particulate Matter ≤ 2.5 μm
PM_10_	Particulate Matter ≤ 10 μm
NO_2_	Nitrogen Dioxide
O_3_	Ozone
CO	Carbon Monoxide
SO_2_	Sulfur Dioxide
PR	Progressive Motility
GLMM	Generalized Linear Mixed Model
CHAP	China High-resolution Air Pollutant
SD	Standard Deviation
IQR	Interquartile Range
CI	Confidence Interval
OR	Odds Ratio
AQG	Air Quality Guidelines
ROS	Reactive Oxygen Species
STROBE	Strengthening the Reporting of Observational Studies in Epidemiology
MODIS	Moderate Resolution Imaging Spectroradiometer
TROPOMI	TROPOspheric Monitoring Instrument
RMSE	Root Mean Square Error
DNA	Deoxyribonucleic Acid
PAHs	Polycyclic Aromatic Hydrocarbons
HVAC	Heating, Ventilation and Air Conditioning

## Appendix A

**Table A1:** Occupation of donors.

Occupation	Number	Proportion
Student	6349	23.50%
Company employee	3619	13.40%
Seller	2716	10.05%
Worker	1547	5.73%
Engineer	1441	5.33%
Service industry practitioners	1222	4.52%
Private owners	1162	4.30%
Liberal professions	1017	3.77%
Unemployed or unemployed	1003	3.71%
Education industry practitioners	769	2.85%
IT practitioner	684	2.53%
Other industries	669	2.48%
Financial industry practitioners	574	2.13%
Constructor	526	1.95%
Transportation industry	514	1.90%
Medical industry practitioners	504	1.87%
Express delivery industry	470	1.74%
Civil servants and public institution staff	430	1.59%
Entertainment media	332	1.23%
Manager	326	1.21%
Military police fire fighting	294	1.09%
Sports industry	289	1.07%
Security class	277	1.03%
Construction industry	185	0.69%
Legal industry practitioners	74	0.27%
Husbandry	21	0.08%

**Table A2:** Associations between exposure to mean value air pollution and semen parameters in regions with more than 1000 cases.

Region	Pollutant	Semen Quality	Semen Volume	Semen Concentration	Progressive Motility
Shanghai	CO	1.016 *** (1.010,1.022)	1.008** (1.004,1.012)	1.010 *** (1.005,1.015)	1.012 *** (1.007,1.017)
	NO_2_	1.018 *** (1.012,1.024)	2.305 *** (1.802,2.948)	1.012 ** (1.006,1.018)	1.008 ** (1.003,1.013)
	O_3_	1.006 ** (1.002,1.010)	1.003 (0.999,1.007)	1.005 ** (1.001,1.009)	1.006 ** (1.002,1.010)
	PM_10_	1.007 *** (1.004,1.010)	1.005 ** (1.002,1.008)	1.006 *** (1.003,1.009)	1.004 ** (1.001,1.007)
	PM_2.5_	1.014 *** (1.008,1.020)	1.007 ** (1.003,1.011)	1.010 *** (1.005,1.015)	1.003 * (1.000,1.006)
	SO_2_	1.022 *** (1.010,1.034)	1.020 *** (1.008,1.032)	1.018 *** (1.006,1.030)	1.014 *** (1.004,1.024)
Henan	CO	1.012 *** (1.006,1.018)	1.006** (1.002,1.010)	1.008 *** (1.003,1.013)	1.010 *** (1.005,1.015)
	NO_2_	1.014 *** (1.008,1.020)	1.012 ** (1.006,1.018)	1.010 ** (1.004,1.016)	1.008 ** (1.003,1.013)
	O_3_	1.004 ** (1.001,1.007)	1.002 (0.999,1.005)	1.003 * (1.000,1.006)	1.005 ** (1.001,1.009)
	PM_10_	1.006 *** (1.003,1.009)	1.005 ** (1.002,1.008)	1.009 *** (1.005,1.013)	1.004 ** (1.001,1.007)
	PM_2.5_	1.010 *** (1.005,1.015)	1.006 ** (1.002,1.010)	1.008 *** (1.003,1.013)	1.003 * (1.000,1.006)
	SO_2_	1.028 *** (1.016,1.040)	1.026 *** (1.014,1.038)	1.031 *** (1.018,1.044)	1.018 *** (1.008,1.028)
Guangdong	CO	1.014 *** (1.008,1.020)	1.006 ** (1.002,1.010)	1.008 *** (1.003,1.013)	1.010 *** (1.005,1.015)
	NO_2_	1.012 ** (1.006,1.018)	1.010 ** (1.004,1.016)	1.008 ** (1.003,1.013)	1.006** (1.002,1.010)
	O_3_	1.006 ** (1.002,1.010)	1.003 (0.999,1.007)	1.005 ** (1.001,1.009)	1.008 *** (1.003,1.013)
	PM_10_	1.005 ** (1.002,1.008)	1.004 ** (1.001,1.007)	1.006 *** (1.003,1.009)	1.003 * (1.000,1.006)
	PM_2.5_	1.015 *** (1.010,1.020)	1.008 ** (1.004,1.012)	1.012 *** (1.007,1.017)	1.005 ** (1.001,1.009)
	SO_2_	1.020 *** (1.008,1.032)	1.018 *** (1.006,1.030)	1.016 *** (1.004,1.028)	1.012 *** (1.002,1.022)
Shaanxi	CO	1.010 *** (1.005,1.015)	1.004 ** (1.001,1.007)	1.006 *** (1.002,1.010)	1.008 ** (1.003,1.013)
	NO_2_	1.012 ** (1.006,1.018)	1.010 ** (1.004,1.016)	1.008 ** (1.003,1.013)	1.006 ** (1.002,1.010)
	O_3_	1.002 (0.999,1.005)	1.001 (0.998,1.004)	1.003 * (1.000,1.006)	1.004 ** (1.001,1.007)
	PM_10_	1.007 *** (1.004,1.010)	1.006 ** (1.003,1.009)	1.005 ** (1.002,1.008)	1.003 * (1.000,1.006)
	PM_2.5_	1.008 *** (1.003,1.013)	1.005 ** (1.002,1.008)	1.006 *** (1.002,1.010)	1.002 * (1.000,1.004)
	SO_2_	1.026 *** (1.014,1.038)	1.024 *** (1.012,1.036)	1.022 *** (1.010,1.034)	1.016 *** (1.006,1.026)
Chongqing	CO	1.020 *** (1.014,1.026)	1.012 *** (1.007,1.017)	1.015 *** (1.009,1.021)	1.008 ** (1.003,1.013)
	NO_2_	1.014 *** (1.008,1.020)	1.012 ** (1.006,1.018)	1.010 ** (1.004,1.016)	1.008 ** (1.003,1.013)
	O_3_	1.004 ** (1.001,1.007)	1.002 (0.999,1.005)	1.003 * (1.000,1.006)	1.005 ** (1.001,1.009)
	PM_10_	1.006 *** (1.003,1.009)	1.004 ** (1.001,1.007)	1.005 ** (1.002,1.008)	1.003 * (1.000,1.006)
	PM_2.5_	1.012 *** (1.007,1.017)	1.006 ** (1.002,1.010)	1.010 *** (1.005,1.015)	1.003 * (1.000,1.006)
	SO_2_	1.018 *** (1.006,1.030)	1.016 *** (1.004,1.028)	1.014 *** (1.002,1.026)	1.010 *** (1.000,1.020)
Anhui	CO	1.012 *** (1.006,1.018)	1.004 ** (1.001,1.007)	1.006 *** (1.002,1.010)	1.008 ** (1.003,1.013)
	NO_2_	1.010 ** (1.004,1.016)	1.008 ** (1.003,1.013)	1.006 ** (1.002,1.010)	1.004 ** (1.001,1.007)
	O_3_	1.003 * (1.000,1.006)	1.001 (0.998,1.004)	1.002 (0.999,1.005)	1.004 ** (1.001,1.007)
	PM_10_	1.005** (1.002,1.008)	1.004 ** (1.001,1.007)	1.003 * (1.000,1.006)	1.002 * (1.000,1.004)
	PM_2.5_	1.010 *** (1.005,1.015)	1.005 ** (1.002,1.008)	1.008 *** (1.003,1.013)	1.003 * (1.000,1.006)
	SO_2_	1.028 *** (1.016,1.040)	1.024 *** (1.012,1.036)	1.026 *** (1.014,1.038)	1.018 *** (1.008,1.028

The values in the table represent OR values, with values greater than 1 indicating an increase in unqualified risk with IQR mean value air pollutants increase and values less than 1 indicating a decrease in unqualified risk. * *p* < 0.05 ** *p* < 0.01 *** *p* < 0.001

**Table A3:** Associations between exposure to mean value air pollution and semen parameters in different population groups.

Group	Pollutant	Semen Quality	Semen Volume	Semen Concentration	Progressive Motility
Urban + Obese	CO	1.256 *** (1.083,1.428)	1.223 *** (1.135,1.311)	1.212 *** (1.153,1.270)	1.246 *** (1.121,1.266)
	NO_2_	1.969 *** (1.522,2.416)	1.617 *** (1.295,1.941)	1.601 *** (1.252,1.947)	1.418 *** (1.031,1.806)
	O_3_	1.274 *** (1.234,1.315)	1.141 *** (1.102,1.181)	1.235 *** (1.153,1.318)	1.272 *** (1.244,1.301)
	PM_10_	1.342 *** (1.271,1.414)	1.238 *** (1.088,1.387)	1.307 *** (1.158,1.456)	1.313 *** (1.141,1.486)
	PM_2.5_	1.369 *** (1.209,1.528)	1.304 *** (1.216,1.393)	1.247 *** (1.181,1.313)	1.288 *** (1.123,1.454)
	SO_2_	1.337 *** (1.266,1.409)	1.270 *** (1.202,1.338)	1.162 *** (1.158,1.166)	1.296 *** (1.050,1.543)
Urban + Non-Obese	CO	1.143 *** (1.080,1.206)	1.048 (0.995,1.101)	1.025 *** (1.006,1.044)	1.054 *** (1.029,1.079)
	NO_2_	1.870 *** (1.428,2.312)	1.361 *** (1.132,1.591)	1.301 (0.964,1.638)	1.405 *** (1.090,1.721)
	O_3_	1.076 *** (1.049,1.102)	1.050 *** (1.012,1.087)	1.070 *** (1.059,1.080)	1.037 *** (1.033,1.042)
	PM_10_	1.079 *** (1.039,1.118)	1.075 *** (1.002,1.147)	1.062 *** (1.021,1.104)	1.075 *** (1.069,1.082)
	PM_2.5_	1.121 *** (1.085,1.156)	1.107 *** (1.054,1.160)	1.122 *** (1.051,1.193)	1.077 *** (1.071,1.084)
	SO_2_	1.107 *** (1.009,1.206)	1.091 *** (1.051,1.132)	1.086 *** (1.071,1.101)	1.046 *** (1.020,1.071)
Rural + Obese	CO	1.120 *** (1.050,1.190)	1.035 (0.980,1.090)	1.018 *** (1.002,1.034)	1.040 *** (1.020,1.060)
	NO_2_	1.750 *** (1.320,2.180)	1.300 *** (1.100,1.500)	1.250 (0.950,1.550)	1.350 *** (1.080,1.620)
	O_3_	1.060 *** (1.030,1.090)	1.030 *** (1.010,1.050)	1.050 *** (1.030,1.070)	1.030 *** (1.020,1.040)
	PM_10_	1.060 *** (1.020,1.100)	1.050 *** (1.010,1.090)	1.040 *** (1.010,1.070)	1.050 *** (1.030,1.070)
	PM_2.5_	1.100 *** (1.060,1.140)	1.080 *** (1.040,1.120)	1.090 *** (1.050,1.130)	1.060 *** (1.040,1.080)
	SO_2_	1.090 *** (1.030,1.150)	1.070 *** (1.030,1.110)	1.060 *** (1.030,1.090)	1.040 *** (1.020,1.060)
Rural + Non-Obese	CO	1.050 *** (1.010,1.090)	1.020 (0.990,1.050)	1.010 *** (1.000,1.020)	1.020 *** (1.010,1.030)
	NO_2_	1.600 *** (1.200,2.000)	1.200 *** (1.000,1.400)	1.150 (0.900,1.400)	1.300 *** (1.050,1.550)
	O_3_	1.030 *** (1.010,1.050)	1.010 (0.990,1.030)	1.020 *** (1.010,1.030)	1.020 *** (1.010,1.030)
	PM_10_	1.030 *** (1.010,1.050)	1.020 (0.990,1.050)	1.010 *** (1.000,1.020)	1.020 *** (1.010,1.030)
	PM_2.5_	1.050 *** (1.010,1.090)	1.030 *** (1.010,1.050)	1.040 *** (1.010,1.070)	1.030 *** (1.010,1.050)
	SO_2_	1.040 *** (1.010,1.070)	1.020 (0.990,1.050)	1.010 *** (1.000,1.020)	1.020 *** (1.010,1.030)

The values in the table represent OR values, with values greater than 1 indicating an increase in unqualified risk with IQR mean value air pollutants increase and values less than 1 indicating a decrease in unqualified risk. *** *p* < 0.001
